# Insecticide resistance mediated by an exon skipping event

**DOI:** 10.1111/mec.13882

**Published:** 2016-11-02

**Authors:** Madeleine Berger, Alin Mirel Puinean, Emma Randall, Christoph T. Zimmer, Wellington M. Silva, Pablo Bielza, Linda M. Field, David Hughes, Ian Mellor, Keywan Hassani‐Pak, Herbert A. A. Siqueira, Martin S. Williamson, Chris Bass

**Affiliations:** ^1^ Rothamsted ResearchHarpendenAL5 2JQUK; ^2^ School of Life SciencesUniversity of NottinghamNottinghamNG7 2RDUK; ^3^ College of Life and Environmental SciencesBiosciencesUniversity of ExeterPenryn CampusPenrynCornwallTR10 9FEUK; ^4^ Departamento de Agronomia—(Entomologia)Universidade Federal Rural de Pernambuco52171‐900RecifePEBrazil; ^5^ Departamento de Producción VegetalUniversidad Politécnica de CartagenaPaseo Alfonso XIII 48Cartagena30203Spain

**Keywords:** alternative splicing, nicotinic acetylcholine receptor, spinosad, *Tuta absoluta*

## Abstract

Many genes increase coding capacity by alternate exon usage. The gene encoding the insect nicotinic acetylcholine receptor (nAChR) α6 subunit, target of the bio‐insecticide spinosad, is one example of this and expands protein diversity via alternative splicing of mutually exclusive exons. Here, we show that spinosad resistance in the tomato leaf miner, *Tuta absoluta* is associated with aberrant regulation of splicing of *Ta*α*6* resulting in a novel form of insecticide resistance mediated by exon skipping. Sequencing of the α6 subunit cDNA from spinosad selected and unselected strains of *T. absoluta* revealed all *Ta*α*6* transcripts of the selected strain were devoid of exon 3, with comparison of genomic DNA and mRNA revealing this is a result of exon skipping. Exon skipping cosegregated with spinosad resistance in survival bioassays, and functional characterization of this alteration using modified human nAChR α7, a model of insect α6, demonstrated that exon 3 is essential for receptor function and hence spinosad sensitivity. DNA and RNA sequencing analyses suggested that exon skipping did not result from genetic alterations in intronic or exonic *cis*‐regulatory elements, but rather was associated with a single epigenetic modification downstream of exon 3a, and quantitative changes in the expression of *trans*‐acting proteins that have known roles in the regulation of alternative splicing. Our results demonstrate that the intrinsic capacity of the α6 gene to generate transcript diversity via alternative splicing can be readily exploited during the evolution of resistance and identifies exon skipping as a molecular alteration conferring insecticide resistance.

## Introduction

Alternative splicing enables a single gene to code for multiple proteins and is thus a key driver of proteome diversity. Despite its importance, the regulation of alternative splicing is complex and not fully understood.

Nicotinic acetylcholine receptors (nAChRs) are neurotransmitter‐gated ion channels that play a vital role in nerve signalling at the postsynaptic membrane of both vertebrates and invertebrates. The nAChRs of insects are a subject of considerable fundamental interest, in part because they exhibit an extraordinary capacity to generate a diversity of mRNA products from single genes as a result of alternate exon splicing, exon exclusion or A‐to‐I pre‐mRNA editing. Indeed, the nAChR Dα6 of *Drosophila melanogaster* is theoretically capable of producing >30 000 different subunit variants (Grauso *et al*. 2002). Insect nAChR α6‐containing receptors are also the target of spinosad, an important bio‐insecticide derived from secondary metabolites of the soil bacteria *Saccharopolyspora spinosa*. Several insect species have developed resistance to this compound and a range of genetic alterations have been described in the α6 subunit of spinosad resistant strains including point mutations (Puinean *et al*. 2013; Bao *et al*. 2014) and other, more profound, alterations that result in truncated nonfunctional proteins (Baxter *et al*. 2010; Rinkevich *et al*. 2010; Hsu *et al*. 2012). Interestingly, such loss‐of‐function mutations do not result in catastrophic loss of fitness as insects without a functional copy of the α6 gene are still viable (Perry *et al*. 2007).

*Tuta absoluta*, the tomato leaf miner, is an economically important pest of tomatoes which, if not controlled, can cause up to 100% yield loss (Desneux *et al*. 2010). Native to South America, this species invaded Europe in 2006 and has since spread to more than thirty European, African and Asian countries (Desneux *et al*. 2011). Spinosad is one of the most important compounds used to control *T. absoluta;* however, its intensive use has now led to reports of resistance (Campos *et al*. 2015).

In the present study, we report the discovery of a novel mechanism of spinosad resistance in *T. absoluta* that exploits the intrinsic capacity of the nAChR α6 gene to generate transcript diversity via alternative splicing and describe an example of insecticide resistance mediated by an exon skipping event.

## Materials and Methods

### Insect strains

Three populations of *Tuta absoluta* (TA1, TA3 and TA4) were collected in 2010 from tomato fields in Spain (Murcia), Italy (Sicily) and Portugal (Lisboa), respectively. The strain GA is a mixed field population collected in 2008 from different areas of Brazil (São Paulo and Minas Gerais states) and reared since then without insecticide selection. The Spin‐parent population was collected from Cilha Queimada, Alcochete, Portugal, in 2012 after reported control failure using spinosad (Spin). The SpinSel population was selected by placing larvae (*n* > 100) on spinosad‐coated tomato leaves for 72 h and taking the survivors to the next generation. Insects were selected for 11 generations, the concentration of spinosad increasing with time to a final concentration of 120 mg/L. All strains were reared on tomato under conditions of 26 °C temperature and 16‐h light.

### Insecticide bioassays

Leaf‐dip bioassays were performed using the procedure outlined in the Insecticide Resistance Action Committee (IRAC) susceptibility Test method 22 (http://www.irac-online.org/content/uploads/Method_022_Tuta.pdf). For synergist bioassays, larvae were exposed to piperonyl butoxide (PBO), diluted with acetone to a concentration of 75 mg/L, for 2 h prior to leaf‐dip bioassay. A range of PBO doses were trialled in preliminary experiments and this was the highest PBO dose which did not cause control mortality. Probit analysis and the calculation of LC_50_ values were performed with GenStat 15th Edition (Payne *et al*. 2011).

### Amplification of the nAChR alpha six subunit from cDNA

Total RNA was extracted from three pools of 10–20 larvae using the ISOLATE II RNA Mini Kit (Bioline) and reverse‐transcribed to cDNA using Superscript III reverse transcriptase (Invitrogen). DreamTaq green (Thermo Fisher Scientific) was used in nested PCR to amplify cDNA using the primers listed in Table S7 (Supporting information). Rapid amplification of cDNA ends (RACE) using the SMARTerTM RACE cDNA Amplification kit (Clontech Laboratories) was used to obtain the 5′ end of *Ta*α*6*. The StrataClone PCR cloning kit (Agilent Genomics) was used to clone PCR products as required.

### Association studies

To examine the association of exon skipping and spinosad resistance, 50 male pupae of the SpinSel strain were collected and placed in a cage containing a tomato plant with 50 female pupae of the Spin strain. Reciprocal crosses were set up in a second cage in the same way. F1 adults were then pooled and allowed to randomly mate to generate F2 larvae. At the L2 stage, approximately eight larvae were placed on a single tomato leaf treated (dipped) with 120 mg/L spinosad, with a total of 15 replicates carried out. Controls (four replicates) comprised L2 larvae placed on single leaves dipped in diluent minus spinosad. Mortality was scored at 72 h as above. Larvae that survived spinosad exposure and control (those exposed to diluent minus spinosad) larvae were snap‐frozen in liquid nitrogen and RNA extracted and cDNA synthesized as above. To genotype individual larvae, the region of the *Ta*α*6* gene encompassing exon 3 was PCR‐amplified as above and PCR products were direct sequenced using the primers listed in Table S7 (Supporting information). The significance of the association between exon skipping and survival to the discriminating dose of spinosad was examined using Fisher's exact test and quantified by calculating the odds ratio.

### PCR and sequencing of the Taα6 exon 3 cluster from genomic DNA

Genomic DNA was extracted from pools of 10–20 larvae using the DNeasy^®^ Plant Mini Kit (Qiagen). The Universal GenomeWalker™ 2.0 kit (Clontech Laboratories) was used to obtain sequence of the large intron downstream of exon 3a and long PCR mix (Thermo Fisher Scientific) was used to amplify genomic DNA. After this initial characterization, a 4000‐bp region encompassing and flanking exons 3a and 3b was sequence characterized from the Spin, SpinSel and TA4 strains using the primers listed in Table S7 (Supporting information). PCR products were direct sequenced in the first instance and cloned and sequenced using the StrataClone PCR cloning kit (Agilent Genomics) if direct sequencing failed to provide clean sequence data.

### Excision of exon 3 from human nAChRa7

Functional expression of insect only nAChRs in heterologous systems has proven technically difficult. In contrast, the vertebrate nAChR α7 readily expresses as a functional receptor in vitro and so human nAChR α7 in pSP64GL (Broadbent *et al*. 2011) was used to study the effect of exon 3 skipping on receptor functionality. Two BglII sites were introduced into *h*α*7* flanking exon 3 using the QuikChange II XL site‐directed mutagenesis kit (Agilent Technologies). Exon 3 was excised and plasmid DNA treated with Mung bean nuclease (New England Biolabs) to remove the 5′ and 3′ overhangs prior to religation using T4 ligase. The absence of exon 3 and the preservation of the reading frame were verified by nucleotide sequencing (Eurofins Genomics).

### *Xenopus* sp. oocytes electrophysiology

Capped RNA was synthesized using the mMessage mMachine SP6 RNA transcription kit (Life Technologies) following the manufacturer's protocol. *Xenopus* sp. oocytes (stages V–VI) purchased from the European Xenopus Resource Centre, University of Portsmouth, were treated with 2 mg/mL collagenase (Sigma‐Aldrich) for 30 min in calcium‐free Barth's solution (NaCl 88 mm, Tris‐HCl 15 mm, NaHCO_3_ 2.4 mm, MgCl_2_ 0.82 mm, KCl 1 mm, pH 7.5) followed by three rinses with clean Barth's solution. Following manual defolliculation, the oocytes were injected with cRNA (25 ng/oocyte) using a Drummond variable volume microinjector. The oocytes were incubated at 18 °C in calcium‐containing (77 mm) Barth's solution with 4 μg/mL kanamycin, 50 μg/mL tetracycline, 100 U/mL penicillin and 100 μg/mL streptomycin. Two‐electrode voltage‐clamp electrophysiology recordings were performed 3–5 days after injection when the oocytes, continuously perfused with frog Ringer's solution (NaCl 115 mm, KCl 2.5 mm, CaCl_2_ 1.8 mm and HEPES 10 mm), were challenged with various concentrations of ligands freshly prepared in frog Ringer's solution. The current required to maintain the membrane potential at −60 mV following drug application was measured.

### RNAseq

To generate a reference transcriptome for *T. absoluta*, RNA was extracted from eggs, larvae, pupae and adults of the TA1 strain using the Bioline Isolate II RNA mini kit (Bioline Reagents Ltd., UK). RNA was pooled and sent to Eurofins Genomics, Germany, for preparation of a random‐primed normalized cDNA library (with an insert size of 150–450 bp) to ensure the detection of transcripts expressed at even low levels. This was sequenced to high coverage on a single lane of an Illumina HiSeq 2000 using 100‐bp paired‐end sequencing. To identify changes in gene expression between the Spin and SpinSel strains, RNA was extracted from three replicates of 10 larvae from each as above, sent to The Genome Analysis CentreTM (TGAC) and multiplexed for sequencing on one lane of an Illumina HiSeq 2000 using 100‐bp paired‐end sequencing. FastQC was used to check the quality of the raw reads obtained (Andrews 2010) prior to de novo assembly using Trinity (release trinityrnaseq_r2013_08_14) (Grabherr *et al*. 2011) as no reference genome exists for *T. absoluta*. Two assemblies were performed: assembly 1 combined RNAseq data from Spin and SpinSel and assembly 2 combined data from TA1, Spin and SpinSel. Contigs obtained were annotated using Blast2Go (Conesa *et al*. 2005). Bowtie implemented in Trinity was used to map raw reads to assembled transcriptomes (Langmead *et al*. 2009), and the rsem software used to estimate gene expression for each biological replicate (Li & Dewey 2011). edger (version 3.14.0) and deseq2 (version 1.12.4) (Robinson *et al*. 2010; Love *et al*. 2014) were used to identify differentially expressed genes using default settings and a corrected *P* value cut‐off of 0.05. Both packages use the negative binomial model for analysing RNAseq count data but differ in their estimation of gene dispersal and genes were only considered differentially expressed if identified as so using both methods.

### Quantitative RT–PCR

Quantitative PCR was carried out using previously described (Bass *et al*. 2013) methods and the primers listed in Table S7 (Supporting information). Data were analysed according to the ΔΔ*C*_T_ method (Pfaffl 2001), using the geometric mean of two selected housekeeping genes (elongation factor delta and eukaryotic initiation factor 5) for normalization according to the strategy described previously (Vandesompele *et al*. 2002).

### Bisulphite PCR and sequencing of the Taα6 exon 3 cluster

Genomic DNA was extracted from the Spin, SpinSel and TA4 strains as detailed above and sent to Zymo Research for bisulphite conversion, PCR and sequencing. DNA was bisulphite converted using the EZ DNA Methylation‐LightningTM Kit (Zymo Research) according to the manufacturer's instructions. A total of 30 amplicons in the case of the SpinSel and TA4 strains and 26 amplicons in the case of the Spin strain, encompassing a 4000‐bp region flanking exons 3a and 3b, were PCR‐amplified using primers listed in Table S8 (Supporting information). Products were barcoded for sequencing on an Illumina MiSeq with >350 000 read pairs obtained for each strain. Reads were aligned to the reference and methylation called at CpG sites using Bismark (Krueger & Andrews 2011). Fisher's exact test was used to identify statistically significant differences in methylation at CpG sites between SpinSel and TA4 and between SpinSel and Spin. To identify sites associated with exon skipping, we asked whether CpG sites differentially methylated (>10% methylation difference and *P* < 0.05) between SpinSel and TA4 are also differentially methylated (and in the same direction) between SpinSel and Spin.

## Results and discussion

### Resistance to spinosad in the SpinSel strain of *Tuta absoluta*

A population of *Tuta absoluta* was collected from an open‐field tomato crop in Portugal after reports of spinosad control failure. Full dose–response bioassays on larvae of this strain ‘Spin‐parent’ revealed a moderate level of resistance (~eightfold) compared to the most susceptible laboratory strain, ‘TA3’ (Table 1). Selection of Spin‐parent with spinosad for 11 generations resulted in the ‘SpinSel’ population which exhibited 277‐fold resistance compared to TA3 (Table 1). An unselected version of Spin‐parent that was not exposed to insecticide for 11 generations ‘Spin’ showed a decrease in resistance of ~fivefold (Table 1). The resistance ratio of the selected and unselected strains at this point was 160‐fold.

**Table 1:** Relative toxicity of spinosad to several strains of *Tuta absoluta*. CI, confidence interval. PBO: treated with 75 mg/L piperonyl butoxide (PBO) for 2 h prior to insecticide treatment

Population	LC_50_ (mg/L)	95% CI
Spin‐parent (F3–F4)	14.9	8.3–23.5
Spin (F20–F21)	3.1	1.3–5.3
SpinSel	498.6	259.3–1105.8
SpinSel (PBO)	431.5	100–866.9
TA1	5.2	3.1–7.3
TA3	1.8	1–2.8
TA4	6.1	3.7–9.6
GA	3	1.8–4.4

To determine whether the resistance of the SpinSel strain was more likely to be conferred by a target‐site or metabolic mechanism, bioassays were conducted on larvae using a pretreatment with piperonyl butoxide (PBO). PBO was selected as an inhibitor of cytochrome P450s and esterases as previous biochemical characterization of spinosad resistance in *T. absoluta* have implicated these enzyme systems in resistance (Reyes *et al*. 2012). No significant differences were observed between SpinSel larvae treated with PBO and untreated controls (Table 1), suggesting resistance was primarily mediated by a target‐site mechanism.

### Spinosad resistance in SpinSel is associated with an exon skipping event resulting in altered nAChR α6 transcripts

The spinosad target site, the nAChR α6 subunit, was identified from *T. absoluta* using next‐generation sequencing of a normalized cDNA library prepared from the TA1 strain (susceptible to spinosad) to identify initial nAChR α6 transcripts, followed by PCR and rapid amplification of cDNA ends (RACE) to obtain full‐length cDNA sequences. The complete coding mRNA sequence of the *Ta*α*6* subunit obtained comprises 1530 bp encoding 510 amino acids and shows high levels of sequence identity with other lepidopteran nAChR α6 gene sequences (Fig. S1,S2, Supporting information). As reported previously for other insects, *Ta*α*6* transcripts were identified containing either of the two mutually exclusive exons 3a and 3b and one of three mutually exclusive exons of exon 8 (Fig. S1, Supporting information).

**Figure 1:**
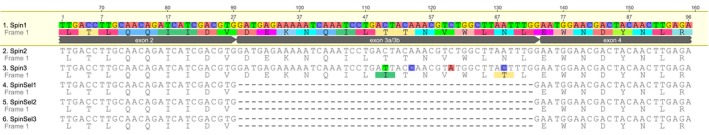
Exon skipping in transcripts of *Ta*α6. Alignment of cDNA sequences of three pooled samples of 10 larvae per replicate of the Spin and SpinSel strain. The region encompassing the 3′ end of exon 2 to the 5′ end of exon 4 is shown. In the case of replicates of the Spin strain double chromatogram peaks at several nucleotide positions across the exon 3 region indicated transcripts with either exons 3a or 3b are expressed, neither exon was observed in sequences from SpinSel. [Colour figure can be viewed at wileyonlinelibrary.com]

Direct sequencing of *Ta*α*6* transcripts from cDNA prepared from three replicate pools of Spin and SpinSel larvae revealed a deletion of 45 bp encoding 15 amino acids (at positions 65–80) in all transcripts obtained from SpinSel. This deletion precisely corresponds to the position and size of exon 3a/3b (Fig. 1). The reading frame of the altered *Ta*α*6* transcripts identified in SpinSel was unaffected by the exclusion of exon 3 and is otherwise identical in amino acid sequence to Spin. To confirm this finding and provide a more accurate estimate of the number of transcripts lacking exon 3a/3b, the relevant region of *Ta*α*6* was PCR‐amplified from cDNA of Spin and SpinSel and cloned and sequenced from ~20 clones (Table S1, Supporting information). In the Spin strain, 50% of clones sequenced represented transcripts with exon 3a, 33% incorporated exon 3b and 17% lacked both exons 3a and 3b, indicating that *Ta*α*6* transcripts modified in this way were almost certainly present in the parental strain at low frequency prior to selection. In contrast, 100% of the clones sequenced from SpinSel lacked exon 3a/3b. A point mutation causing an amino acid substitution (G275E) in exon 9 of *Ta*α*6* has been recently associated with spinosad resistance in populations of *T. absoluta* in Brazil (Silva *et al*. 2016), but this was not observed in either Spin or SpinSel.

**Figure 2:**
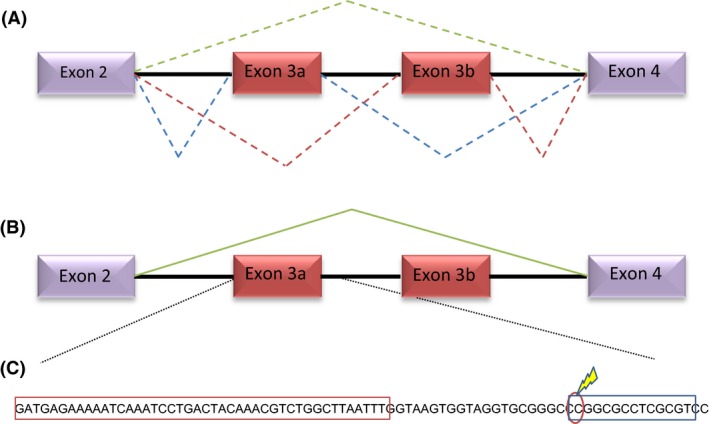
Patterns of splicing and DNA methylation of the *Tuta absoluta* nAChR α6 subunit gene. (A) The spinosad susceptible strain Spin exhibits mutually exclusive splicing of exon 3a/3b (blue and red dashed lines), but a low frequency of transcripts exhibits exon skipping (green dashed line). (B) In the spinosad resistant strain SpinSel, all *T*α*6* transcripts exclude both exons 3a and 3b (green solid line). (C) Nucleotide sequence of exon 3a and immediate region downstream highlighting the position of the single CpG site that is 30% methylated in the SpinSel strain (marked with a lightening bolt). The sequence encoding exon 3a is boxed in red. The region boxed in blue indicates a predicted CTCF binding site. [Colour figure can be viewed at wileyonlinelibrary.com]

A possible explanation for the altered *Ta*α*6* transcripts observed in the SpinSel strain is a molecular alteration that disturbs the alternative splicing of exons 3a and 3b resulting in the exclusion of both exons. In this scenario, both exons would still be present in a genomic context, but a *cis*‐ or *trans*‐acting alteration results in the loss of exon recognition. To confirm this, DNA was extracted from SpinSel larvae and the genomic region encompassing both exons was PCR‐amplified and sequenced. Both exon 3a and exon 3b are present and unaltered in genomic DNA of SpinSel (Fig. S3, Supporting information), indicating that the loss of these exons in mRNA results from exon skipping rather than a deletion of the exons in genomic DNA. Thus, in the Spin strain, most mRNA transcripts contain one or other of the two mutually exclusive exons 3a or 3b depending on which of the two alternate 3′ splice sites upstream of each exon is recognized. In the case of SpinSel transcripts, both exons 3a and 3b are skipped with only the 5′ splice site of the intron downstream of exon 2 and the 3′ splice site in the intron upstream of exon 4 recognized (Fig. 2A,B).

Interestingly, low levels of exon skipping in insect nAChRs appear to be a common post‐transcriptional phenomena and are observed in a wild‐type genetic background. This includes exon 3 where α*6* transcripts missing both isoforms have been identified in silkworm, *Bombyx mori* and red flour beetle, *Tribolium castaneum* (Shao *et al*. 2007; Rinkevich & Scott 2009). In the latter case, exon 3 skipping was observed in α*6* transcripts at a frequency of ~13% (Rinkevich & Scott 2009), and in both cases, exon 3 skipping did not cause a frame shift and transcripts otherwise retained the same nucleotide and amino acid identity as transcripts with exon 3. Exclusion of other exons in alternative nAChR subunit subtypes has also been described in *Drosophila melanogaster* (Sattelle *et al*. 2005). Cloning and sequencing of four additional spinosad susceptible strains of *T. absoluta* also suggested exon skipping exists in *T. absoluta* at low frequency with exon 3 skipping observed in one out of 21 colonies sequenced from these strains (Fig. S4, Supporting information). The functional significance, if any, of these findings is not clear. It has been suggested that truncated nAChR subunits may act as an ‘acetylcholine sponge’ in a manner similar to that of the molluscan ACh‐binding protein (Smit *et al*. 2003). However, nAChR α*6* subunits without exon 3a/3b are missing binding loop D which is crucial for ACh interaction, making this explanation unlikely. Alternatively, studies have suggested such transcripts may regulate the expression of full‐length nAChR transcripts (Garcia‐Guzman *et al*. 1995; Saragoza *et al*. 2003). This includes nAChR transcripts exhibiting exon skipping, as demonstrated by α7 transcripts from bovine adrenal chromaffin cells in which exon 8 is skipped. Such transcripts did not form functional channels when expressed alone but produced dose‐dependent inhibition of alpha 7 homomer expression when co‐injected with the undeleted isoform (Garcia‐Guzman *et al*. 1995).

Regardless, it is clear that the ‘normal’ frequency of exon skipping in *Ta*α*6* has been profoundly altered in the spinosad selected strain of *T. absoluta* as, in contrast to the parental strain, all transcripts were devoid of either form of this exon. Disruption of alternative splicing has been associated with resistance to spinosad previously. In a spinosad resistant strain of diamondback moth, *Plutella xylostella,* collected in Hawaii, a mutation within the ninth intron splice junction of *Px*α*6* resulted in mis‐splicing of transcripts and a predicted protein truncated between the third and fourth transmembrane domains (Baxter *et al*. 2010). Additional splice forms were identified in the resistant strain, all of which introduced in‐frame premature stop codons. A second study on a spinosad resistant *P. xylostella* strain from the same region also identified a range of altered transcripts associated with resistance, including two transcripts expressed at low frequency that showed skipping of exon 3 (Rinkevich *et al*. 2010). However, in both studies, and in contrast to our study, a wide variety of altered transcripts were observed in resistant strains of *P. xylostella* with almost all cases, including those showing exon 3 skipping, introducing premature stop codons.

### Exon skipping cosegregates with spinosad resistance in survival bioassays

To further examine the association between exon skipping and spinosad resistance, the Spin and SpinSel strains were reciprocally crossed and F1 progeny pooled and allowed to mate randomly to generate a hybrid F2 population containing all possible phenotypes/genotypes. A subset of this population was then exposed to a discriminating dose of spinosad for 72 h, which, based on the recessive inheritance of loss‐of‐function mutations in the nAChR α6 gene of other insects (Perry *et al*. 2007), was predicted to kill all but homozygous resistant individuals. A second subset of the population was treated in exactly the same way but exposed to diluent minus insecticide. Across 15 replicates, an average of 34% of the F2 population exposed to spinosad survived exposure to bioassay (Table S2, Supporting information). Of these PCR and sequencing revealed that 10 out of 14 individuals successfully genotyped (71%) completely lacked exon 3 (were homozygous resistant). In contrast, in the unexposed subset of the population, just five out of 25 individuals sequenced (20%) were homozygous for exon 3 skipping. These data reveal a significant (Fisher's exact test *P* = 0.0024, odds ratio 10, 95% confidence intervals 2.19–45.64) association between exon 3 skipping and survival to spinosad exposure supporting a causal role in resistance.

### Exon 3 of the nAChR is essential for receptor function and spinosad sensitivity

**Figure 3:**
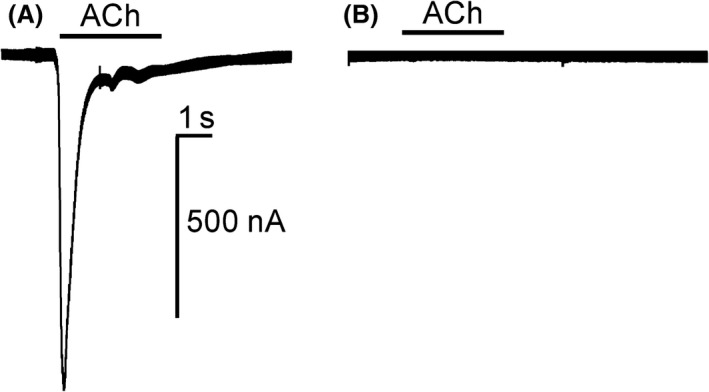
Response of nAChR α7 (A) or nAChR α7^−Exon3^. (B) Expressed in *Xenopus* oocytes to 0.3 mm acetylcholine.

Although the exon skipping event observed in the SpinSel strain does not introduce premature stop codons it would be predicted to have a profound effect on the function of receptors containing this subunit as a critical component of the ACh‐binding site, loop D, would be missing in the resulting protein (Grutter & Changeux 2001). Functional characterization of insect nAChRs has been hampered by the difficulties encountered in expressing them in heterologous systems. However, the vertebrate nAChR α7 readily expresses as a functional receptor and has been successfully used as a model to further our understanding of the interaction of spinosyn insecticides with the receptor (Puinean *et al*. 2013). The functional consequences of exon 3 skipping were therefore examined by excising exon 3 from human nAChR α7 and expressing the modified cRNA in *Xenopus* oocytes alongside wild‐type nAChR α7. The formation of functional receptors was examined by two‐electrode voltage‐clamp recording, challenging the oocytes with a range of acetylcholine concentrations. As shown in Fig. 3, activation of nAChR α7 wild‐type receptors produced a typical fast desensitizing response while nAChR α7^−Exon3^ expressing oocytes did not yield any inward current following ACh application. The loss of activation by ACh observed for the modified human nAChR α7 could be due to either the formation of a receptor lacking the acetylcholine binding site or to the expression of a misfolded subunit that does not get assembled into a functional receptor. In a previous study (Gill *et al*. 2011), the influence of a mutation located close to the ACh‐binding site on the human nAChR α7 was examined and found to cause a significant decrease of ACh activity. However, the same mutation had a limited effect on 4‐(4‐bromophenyl)‐3a,4,5,9b‐tetrahydro‐3H‐cyclopenta[c]quinoline‐8‐sulfonamide (4BP‐TQS), agonist activity, providing strong evidence that the two ligands bind to different sites on the human nAChR α7. Therefore, to determine whether nAChR α7^−Exon3^ subunits still form functional receptors but lack the orthosteric agonist site, the oocytes were tested with 4BP‐TQS. Again, the nAChR α7^−Exon3^ expressing oocytes failed to respond, strongly suggesting that exon 3 skipping produces a nonfunctional receptor that would not be affected by the action of spinosad.

### Exon skipping is not associated with a genetic alteration in an intronic or exonic cis‐regulatory element

The regulation of alternative splicing is complex and highly combinatorial (Keren *et al*. 2010; Fu & Ares 2014). Alternative exons are affected by multiple RNA elements and RNA‐binding proteins that can be both positive and negative acting and are often combined in an intricate regulatory system. A common mechanism of exon skipping in other organisms is the alteration of conserved *cis*‐elements observed at exon–intron junctions such as the splice sites (Ward & Cooper 2010). To explore this possibility, the exonic and flanking intronic sequences of *Ta*α*6* were PCR‐amplified and sequenced from Spin and SpinSel. PCR amplification of the genomic region encompassing exons 2–4 in the SpinSel strain showed exon 3a is flanked by a large upstream intron of >10 kb, the intron between exon 3a and 3b is comparatively smaller at 899 bp, and the intron downstream of exon 3b is 4675 bp in size. The coding sequence of both exon 3a and 3b was identical in the genomic sequence of Spin and SpinSel (Fig. S3, Supporting information) ruling out the possibility of a mutation in an exonic splicing regulator binding site, such as an exon splicing enhancer or silencer (ESE/ESS), which promote exon inclusion or exclusion. The Splicing Regulation Online Graphical Engine (SROOGLE) was used to identify splice/branch sites and polypyrimidine tracts flanking exons 3a and 3b (Schwartz *et al*. 2009). No modification of the 5′ or 3′ splice sites (which are all canonical), or branch sites in these introns was observed when sequences of Spin and SpinSel were compared (Fig. S3, Supporting information). However, a single t/c SNP was observed in the polypyrimidine tract upstream of exon 3a in SpinSel (Fig. S3, Supporting information). In addition to polymorphisms at core splicing elements, we also looked for genetic variation that might affect regulatory intronic sequences that bind RNA‐binding proteins, specifically those acting as positive or negative effectors of splicing such as intronic splicing enhancers and silencers (ISEs and ISSs, respectively). Significant genetic variation (including SNPs and large INDELs) was seen in the intron sequences flanking exons 3a/3b between Spin and SpinSel (see Fig. S3, Supporting information). In the case of the Spin population, which was not selected with insecticide, a much greater degree of heterozygosity was observed which required cloning and sequencing to resolve. Because of the multitude of sequence polymorphisms observed between the principal sequence haplotypes of Spin and SpinSel, it would be difficult to ascertain which, if any, SNPs/INDELs result in modified splicing of exon 3a/3b. To see whether any of the genetic diversity observed was conserved in other *T. absoluta* populations, additional spinosad susceptible strains were sequenced across a 4000‐bp region encompassing and flanking exons 3a and 3b. In the case of one of these strains, ‘TA4′, the primary haplotype observed was 100% identical in sequence to that observed for SpinSel, including at the site of the SNP observed between Spin and SpinSel in the polypyrimidine tract detailed above (see Fig. S3, Supporting information). The TA4 strain is susceptible to spinosad (see Table 1) and analysis of nAChR transcripts of this strain revealed no evidence of exon skipping with 50% of clones containing exon 3a and 50% exon 3b. This finding strongly suggests that the sequence differences observed between the Spin and SpinSel strain around exons 3a and 3b are unlikely to result in exon skipping and hence resistance.

### Exon skipping is associated with an epigenetic modification downstream of exon 3a in a predicted CTCF binding site

Recent reports have provided emerging evidence of a role for epigenetic mechanisms, such as DNA methylation, in regulating alternative RNA splicing (Maor *et al*. 2015). To explore the association of exon skipping with the level of methylation at CpG sites within the exon 3 cluster, we carried out bisulphite PCR and pyrosequencing on genomic DNA extracted from the Spin, SpinSel and TA4 strains. A 4‐kb region encompassing the exon 3 cluster and including 144 unique CpG sites in the TA4 and SpinSel strains and 120 sites in the Spin strain was PCR‐amplified from bisulphite converted DNA with subsequent pyrosequencing providing an average CpG coverage of >500X (Table S3, Supporting information). The number of CpG sites identified in the Spin strain was comparatively lower than in SpinSel/TA4 due to the numerous SNPs and INDELs observed in the primary haplotype of this strain, leading to an overall reduction in the total number of CpG sites. The level of observed methylation at CpG sites over the exon 3 cluster was very low in all three strains. Methylation ratios (the measured number of methylated cytosines divided by total number of cytosines covered at that site) of >10% were observed at just a single CpG site in the SpinSel strain and at four sites in the TA4 and Spin strains (Table S3, Supporting information). Of these, statistically significant differences in methylation between the SpinSel strain and both the TA4 and Spin strains were observed at just a single CpG site (*P* = <0.001, position 2030, Table S3, Supporting information) which exhibited a methylation ratio of 30% in SpinSel vs. 0% and 0.5% in TA4 and Spin, respectively. This CpG site is in intronic sequence 21 bp downstream of exon 3a (Fig. 2C). Methylation downstream of alternative exons has recently been shown to result in exon skipping by inhibiting binding of the DNA‐binding protein CCCTC‐binding factor (CTCF) (Shukla *et al*. 2011). The reduction in CTCF binding releases local RNA polymerase II pausing resulting in reduced exon inclusion levels (Shukla *et al*. 2011). We used the CTCFBSDB 2.0 database (Ziebarth *et al*. 2013) to scan for CTCF core binding sites in and downstream of exon 3a. A predicted match to the CTCF core motif defined by the EMBL_M1 position weight matrices was identified at position +21 to 34 bp downstream of exon 3a (Fig. 2C) with the first nucleotide of this motif corresponding to the CpG site that is methylated in SpinSel. It is therefore feasible that methylation of this CpG site may be inhibiting CTCF binding and so contributing to the lowered inclusion of exon 3a. The effect, if any, of methylation at this site on the inclusion of exon 3b is unclear and it is likely that additional mechanisms, such as alteration in the expression of splice factors, result in the observed skipping of exon 3a/b.

### Exon skipping is associated with altered expression of splice factors

Alternative exons like exon 3a/b in *Ta*α*6* usually have weaker splice sites than constitutive exons and are thus especially reliant on *trans*‐acting splice factor proteins for exon selection (Keren *et al*. 2010). Furthermore, it has been shown that even relatively subtle changes in splice factor protein levels can lead to the deregulation of alternative splicing (Khan *et al*. 2012). To investigate whether the expression of such factors is altered in the SpinSel strain, global gene expression levels in Spin and SpinSel larvae were compared using RNAseq. Since there is no reference genome for *T. absoluta,* two different de novo assemblies of the sequencing reads obtained were performed and these were used as independent references to map reads of Spin and SpinSel in order to assess gene expression. The first assembly (assembly 1) only contained reads from Spin and SpinSel, while the second (assembly 2) combined these reads with those of another strain TA1 which was sequenced previously (see Methods). Assembly statistics are shown in Table S9 (Supporting information). A total of 289 transcripts, that were assigned a functional description by BLAST (see Methods), were identified as significantly differentially expressed (76 overexpressed and 213 underexpressed in SpinSel when compared to Spin) using the Spin/SpinSel assembly as a reference, and a total of 254 annotated transcripts identified as differentially expressed (68 overexpressed and 186 underexpressed) using the Spin/SpinSel/TA1 assembly as a reference (Tables S4/S5, Supporting information).

Interrogation of these gene lists identified a number of transcripts encoding potential splice factor proteins including suppressor of white apricot (SWAP), RNA‐binding protein 1 (RBP1), gem‐associated protein 5 (Gemin5) and u11 u12 small nuclear ribonucleoprotein 48 kDa (Table 2). All of these were significantly downregulated in the SpinSel strain. SWAP and RBP1 belong to the serine/arginine‐rich (SR) family of splicing factors that have been shown to be critically important proteins in exon recognition and the regulation of alternative splicing (Howard & Sanford 2015). Importantly, SR proteins have been shown to play a role in ensuring that 5′ and 3′ splice sites within the same intron are used and thus act to suppress exon skipping (Ibrahim *et al*. 2005). It is feasible therefore that the downregulation of SR proteins in the SpinSel strain may lead to exon skipping as exon 3 exclusion results from the use of 5′ and 3′ splice sites within different introns (the 5′ splice site in the intron downstream of exon 2 in combination with the 3′ splice site upstream of exon 4, see Fig. 2). Gemin5 is a component of the spliceosomal complex that has been shown to influence alternative mRNA splicing (Lee *et al*. 2008). A study of the role of Gemin5 in human cancer cells found that alteration in the expression of this gene caused alternative splicing events in at least 16 genes (Lee *et al*. 2008). In the case of three of these genes, calcium/calmodulin‐dependent protein kinase IV, STK32C and TIE1, the altered splicing event involved exon skipping. In this prior study, exon skipping was associated with the overexpression of Gemin5 and whether downregulation of this gene, as seen in SpinSel, may cause similar splicing abnormalities is unknown. Finally, the u11 u12 small nuclear ribonucleoprotein 48 kDa is part of the minor spliceosome protein complex, involved in 5′ splice site recognition of U12‐type introns (Turunen *et al*. 2013). However, this part of the minor spliceosome protein complex is involved in 5′ splice site recognition of U12‐type introns (Turunen *et al*. 2013) and so its relevance in exon skipping in the SpinSel strain is unclear as the introns flanking exon 3a/3b are of the major U2‐type.

**Table 2:** Transcripts encoding splice factors that are differentially expressed between the Spin and SpinSel strains in RNAseq analysis. Expression differences estimated by DESeq2 are provided as fold changes (FC) and log fold changes (logFC). Adjusted (i.e. corrected) *P* values (padj) are also provided

Contig ID	Description	FC	logFC	padj
comp67725_c1_seq2	u11 u12 small nuclear ribonucleoprotein 48 kDa	0.0485	−4.3659	2.5E‐05
comp62457_c0_seq2	Gem‐associated protein 5‐like	0.0390	−4.6799	3.6E‐05
comp72316_c0_seq4	Protein suppressor of white apricot‐like	0.0187	−5.7401	3.3E‐09
comp150190_c0_seq2	Protein suppressor of white apricot‐like	0.0477	−4.3908	1.2E‐04
comp142486_c0_seq8	RNA‐binding protein 1	0.0482	−4.3749	1.4E‐04
comp142486_c0_seq10	RNA‐binding protein	0.0209	−5.5795	4.3E‐09

**Figure 4:**
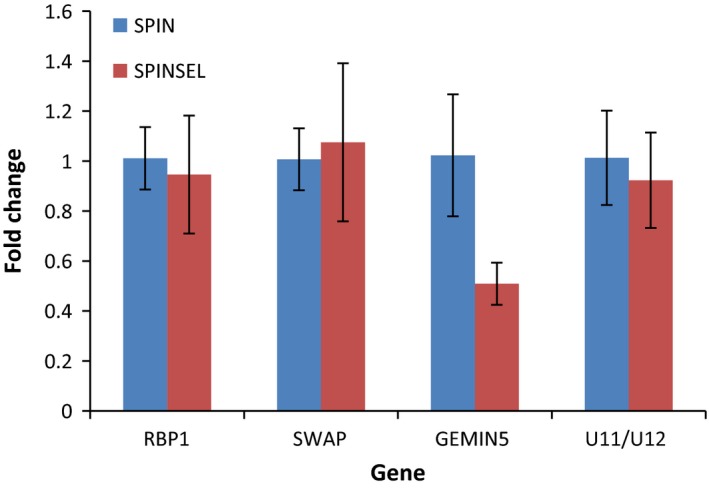
Fold change in expression of genes encoding splice factors in the spinosad resistant SpinSel strain (compared to the susceptible Spin strain) as determined by quantitative PCR. Error bars indicate 95% confidence intervals. [Colour figure can be viewed at wileyonlinelibrary.com]

To validate the results of RNAseq analyses, the expression of the candidate genes identified was examined in Spin and SpinSel by qPCR. Surprisingly, only Gemin5 was confirmed as significantly downregulated in the SpinSel strain (Fig. 4). Further investigation suggested that in the case of SWAP and RBP1, this anomaly may result from mapping reads to a transcriptome assembly (rather than a reference genome). During de novo assembly, Trinity outputs multiple isoforms for a ‘gene’ if evidence exists for alternative splicing (Grabherr *et al*. 2011). Interrogation of the count matrix for each candidate gene revealed evidence that only certain ‘isoforms’ (i.e. specific alternatively spliced transcripts) are differentially expressed. This is illustrated in Table S6 (Supporting information) for the gene SWAP where four isoforms were identified with a SWAP annotation; of these, two were assessed as differentially expressed using an FDR cut‐off of <0.05 (comp150190_c0_seq2 and comp72316_c0_seq4). Both downregulated isoforms contained a 15‐bp INDEL in the 5′ UTR in comparison with the two isoforms which were not differentially expressed (Fig. S5, Supporting information). The functional significance of these different isoforms is not clear; however, several distinct transcripts resulting from alternative splicing have been recorded in other insects for both SWAP and RBP1 that may encode proteins with different functions, or be nonfunctional (Zachar *et al*. 1987; Wang *et al*. 2010).

Beyond splice factors, transcriptome profiling of the Spin and SpinSel strains revealed very few genes overexpressed in the SpinSel strain belonging to the major superfamilies of enzymes frequently implicated in metabolic resistance in insects. For example, no transcripts encoding cytochrome P450s (P450s), carboxylesterases (CEs) or UDP‐glucuronosyltransferase (UGTs) were identified as overexpressed in reads mapped to either assembly 1 or 2 (Table S4/S5, Supporting information). A single transcript encoding a glutathione S‐transferase (GST) (comp155108_c1_seq10) was identified as upregulated (17‐fold by DESeq2) in the SpinSel strain in reads mapped to assembly 2 (Table S5, Supporting information). However, this transcript encodes a conserved GST enzyme belonging to the zeta class that corresponds to GST zeta 1 (GSTZ1), an enzyme catalysing maleylacetoacetate to fumarylacetoacetate in the penultimate step of the phenylalanine/tyrosine degradation pathway (Fernández‐Cañón & Peñalva 1998), and is not known to play a role in the metabolism of xenobiotics. A single transcript (comp62327_c1_seq6) encoding an aldo‐keto reductase, an enzyme family known to play an important role in the phase II detoxification of several drugs and xenobiotics in humans, was identified as overexpressed (7.4‐fold) in the assembly 1 comparison (Table S4, Supporting information); however, an almost identical transcript (comp145080_c0_seq1) of the same gene (99% sequence identity) was identified as downregulated in the assembly 2 comparison (Table S5, Supporting information). Finally, no transcripts with potential roles in phase III metabolism (i.e. ABC transporters) were overexpressed in the SpinSel strain in either comparison. In summary, RNAseq analysis provided little evidence of additional, non‐target‐site, mechanisms of resistance in the SpinSel strain.

## Conclusions

These data describe a form of target‐site resistance mediated by exon skipping. This mechanism of resistance has not been reported in isolation previously as most insecticide target proteins are essential for viability and cannot tolerate such loss‐of‐function alterations. In this instance, because the nAChR α6 subunit is a redundant target site, it is freed from these evolutionary constraints. We believe the exon skipping we have identified derives from a natural alternative splicing event observed at low frequency in insects, with the evolution of resistance an example of adaptation from standing transcriptomic variation. The dramatic increase in frequency of this native exon skipping event in the SpinSel strain clearly provides a selective advantage in the presence of spinosad with the truncated proteins produced nonfunctional and hence unaffected by this insecticide.

The regulation of mutually exclusive exons, like exon 3 of the nAChR α6 gene, involves the complex interplay between nuclear proteins and the *cis*‐regulatory sequences they bind to in pre‐mRNA. Thus, the exon skipping event seen in the SpinSel strain may result from genetic mutations that disrupt *cis*‐regulatory elements such as exonic and intronic splicing regulatory sequences (ESRs/ISRs) or from quantitative or qualitative changes in *trans*‐acting proteins such as splicing factors. In addition, a growing body of work has provided evidence of the importance of epigenetic mechanisms such as changes in DNA methylation in the regulation of alternative splicing. This study provides evidence of a role for the latter processes with exon skipping associated with a change in the level of methylation downstream of exon 3a and the expression of at least one important splice factor. Further detailed exploration of the relative contribution of these mechanisms in exon skipping and insecticide resistance is now required. In this regard, recent advances in (epi)genome editing using the CRISPR/Cas9 system has made it possible to both modify candidate gene expression and carry out epigenetic editing at specific loci (Dominguez *et al*. 2016; Vojta *et al*. 2016).

M.B., M.S.W. and C.B. conceived and designed the study. M.B., A.M.P., E.R., C.T.Z., W.M.S., L.M.F., D.H. and C.B. performed experiments and analysed data. M.B., A.M.P., P.B., L.M.F., D.H., I.M., K.H.P., H.A.A.S., M.S.W. and C.B. wrote the manuscript.

## Data accessibility

Raw Sequence Data obtained in this study has been deposited in the National Center for Biotechnology Information Sequence Read Archive under the following Accession nos.: SRR2846714, SRR2913248, SRR2913250, SRR2913254, SRR2913258, SRR2913261, SRR2913263. Assembled transcriptomes and corresponding annotation files have been deposited in the online Dryad Digital Repository (www.datadryad.org; doi:10.5061/dryad.37p17).

## Supporting information


**Fig. S1** (A) cDNA and predicted protein sequence of the *Tuta absoluta* nAChR α6 subunit (with exon 3a and 8a). (B) Alternative exons 3a and 3b and exons 8a, 8b and 8c of the *Tuta absoluta* nAChR α6 subunit.

**Fig. S2** Comparison of the amino acid sequence of the *Tuta absoluta* α6 subunit with that of *Bombyx mori* (GenBank ABL67934.1) and *Plutella xylostella* (GenBank GU207835.1).

**Fig. S3** Alignment of a 4000 bp genomic sequence encompassing the Taα6 exon 3 cluster from the Spin, SpinSel and TA4 strains.

**Fig. S4** Amino acid alignment of the exon 2–4 region of *Ta*α*6* from 21 sequenced clones generated from four different *Tuta absoluta* spinosad susceptible strains (TA1, TA3, TA4, GA).

**Fig. S5** Alignment of contigs encoding suppressor of white apricot from two de novo *Tuta absoluta* transcriptome assemblies.

**Table S1** Exon three usage in sequenced clones of *Ta*α*6* in the Spin and SpinSel strains.

**Table S2** Larval mortality of F2 hybrids of the Spin and SpinSel strains to a discriminating dose of spinosad (120 mg/L).

**Table S3** Table of CpG sites and corresponding methylation ratios in the exon 3 cluster for the SpinSel, Spin and TA4 *Tuta absoluta* strains as revealed by bisulphite sequencing.

**Table S4** Transcripts identified by RNAseq as significantly differentially expressed between the Spin and SpinSel *Tuta absoluta* strains using assembly one as a reference (see methods).

**Table S5** Transcripts identified by RNAseq as significantly differentially expressed between the Spin and SpinSel *Tuta absoluta* strains using assembly two as a reference (see methods).

**Table S6** Expression of isoforms encoding suppressor of white apricot in the *Tuta absoluta* transcriptome.

**Table S7** Sequences of primers used in this study.

**Table S8** Sequence of primers used for Bisulphite PCR.

**Table S9** Assembly statistics of *Tuta absoluta* transcriptomes.
