# Supplementary material for: Insecticide resistance mediated by an exon skipping event
Source: Mol Ecol. 2016 Nov 2;25(22):5692–704. doi: 10.1111/mec.13882 (PMC5111602; doi:10.1111/mec.13882)

Figure S1. A) cDNA and predicted protein sequence of the *T. absoluta* nAChR α6 subunit (with exon3a and 8a). B) Alternative exons 3a and 3b and exons 8a, 8b and 8c of the *T. absoluta* nAChR α6 subunit.


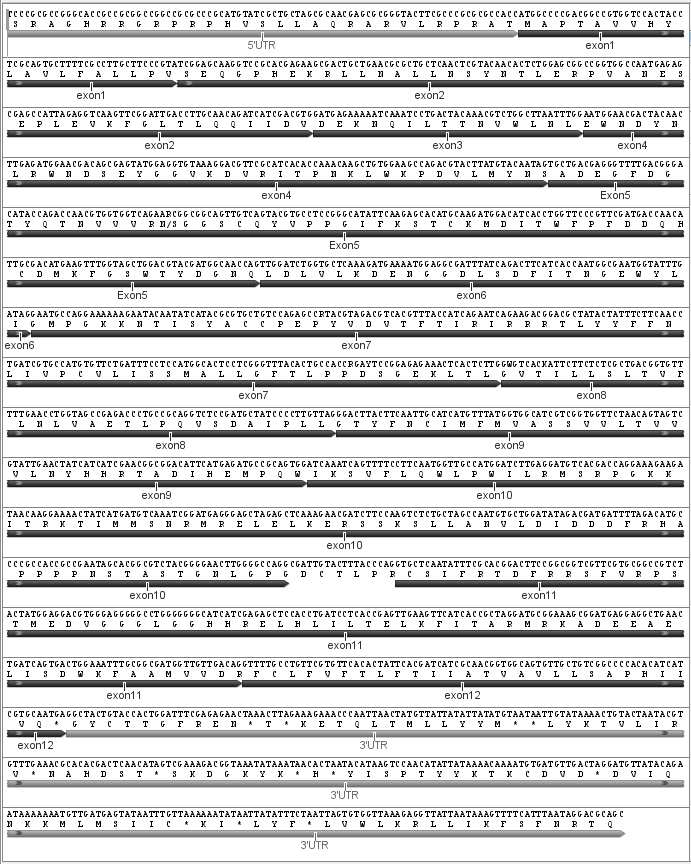


B)


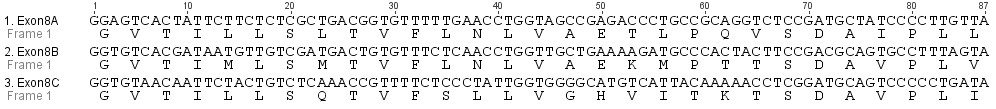

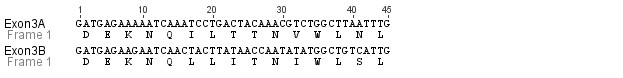

Supplement: Supplementary file 1 — Fig. S1 (A) cDNA and predicted protein sequence of the Tuta absoluta nAChR α6 subunit (with exon 3a and 8a). (B) Alternative exons 3a and 3b and exons 8a, 8b and 8c of the Tuta absoluta nAChR α6 subunit. [file MEC-25-5692-s001.docx]
