# Supplementary material for: Insecticide resistance mediated by an exon skipping event
Source: Mol Ecol. 2016 Nov 2;25(22):5692–704. doi: 10.1111/mec.13882 (PMC5111602; doi:10.1111/mec.13882)

Figure S2. Comparison of the amino acid sequence of the *T. absoluta* α6 subunit with that of *B. mori* (GenBank ABL67934.1) and *P. xylostella* (GenBank GU207835.1). Dots indicate conserved sequence.


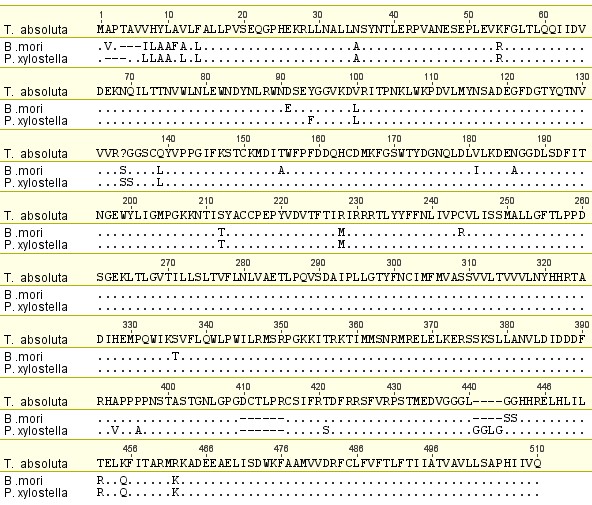

Supplement: Supplementary file 2 — Fig. S2 Comparison of the amino acid sequence of the Tuta absoluta α6 subunit with that of Bombyx mori (GenBank ABL67934.1) and Plutella xylostella (GenBank GU207835.1). [file MEC-25-5692-s002.docx]
