# Supplementary material for: Insecticide resistance mediated by an exon skipping event
Source: Mol Ecol. 2016 Nov 2;25(22):5692–704. doi: 10.1111/mec.13882 (PMC5111602; doi:10.1111/mec.13882)

Figure S3. Alignment of a 4000bp genomic sequence encompassing the Taα6 exon 3 cluster from the Spin, SpinSel and TA4 strains. In each case the consensus sequence for each strain is shown. Nucleotides which differ between populations are highlighted. Exons 3A and 3B are indicated. Canonical dinucleotides for donor and acceptor sites (AG and GT) are indicated by yellow boxes.


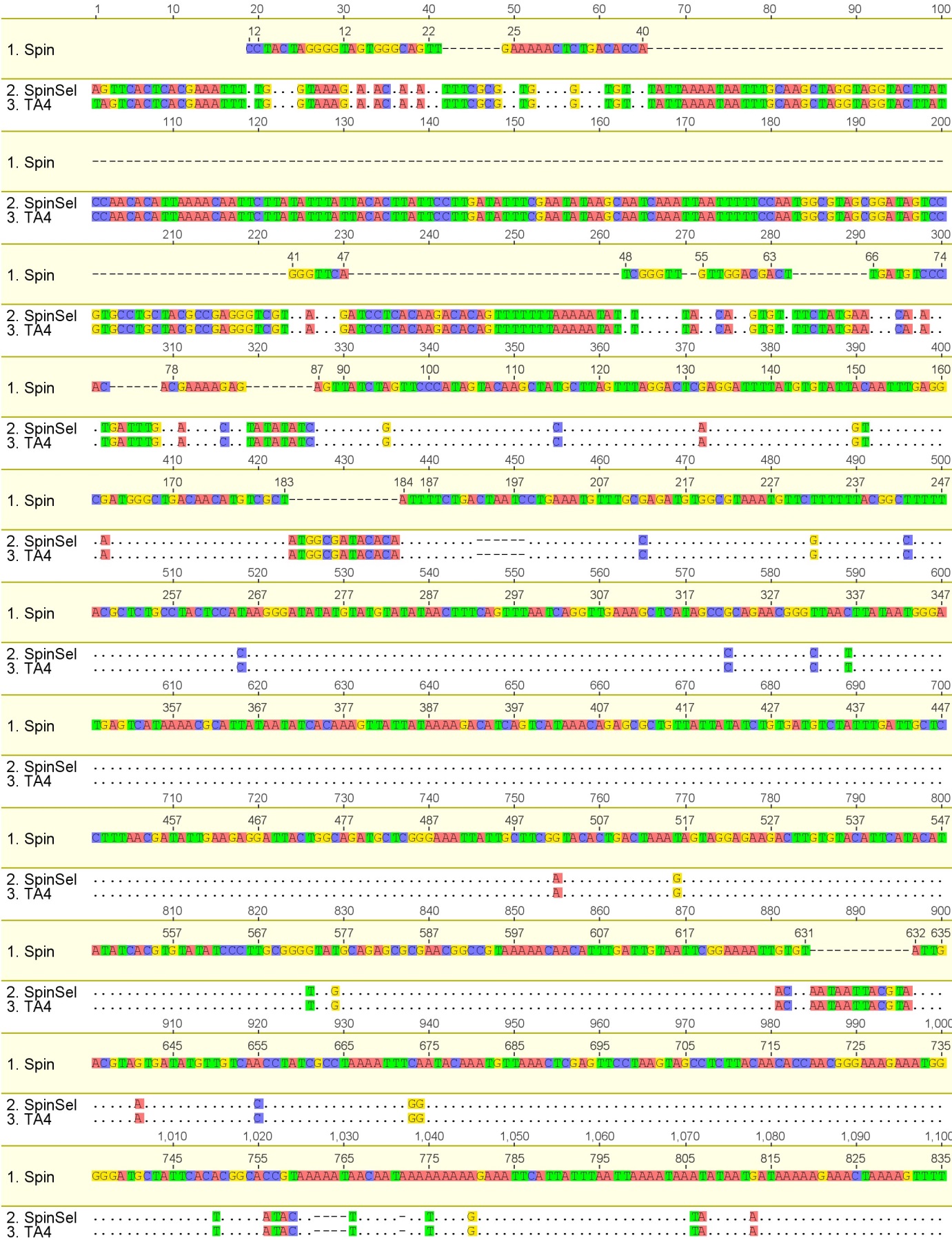


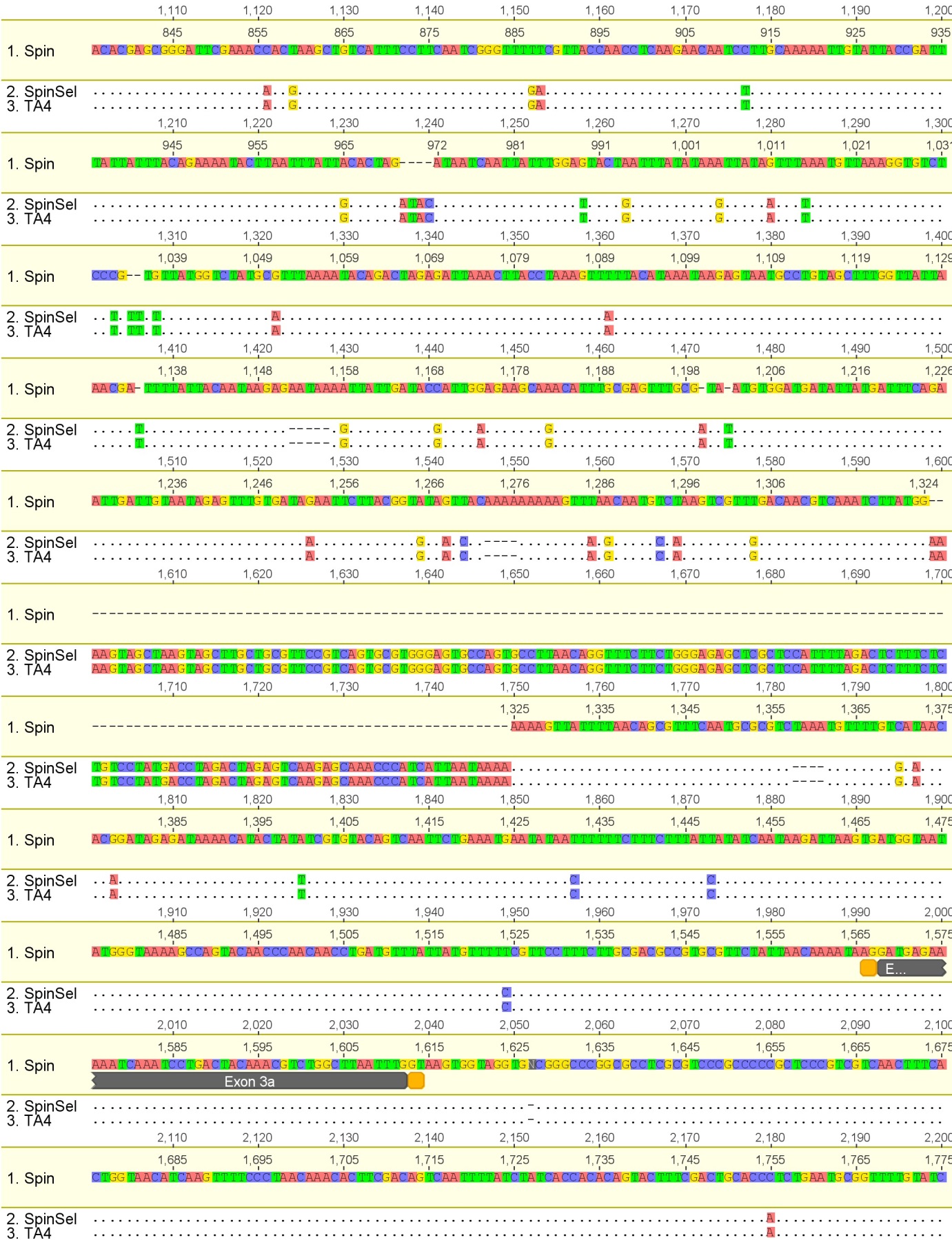


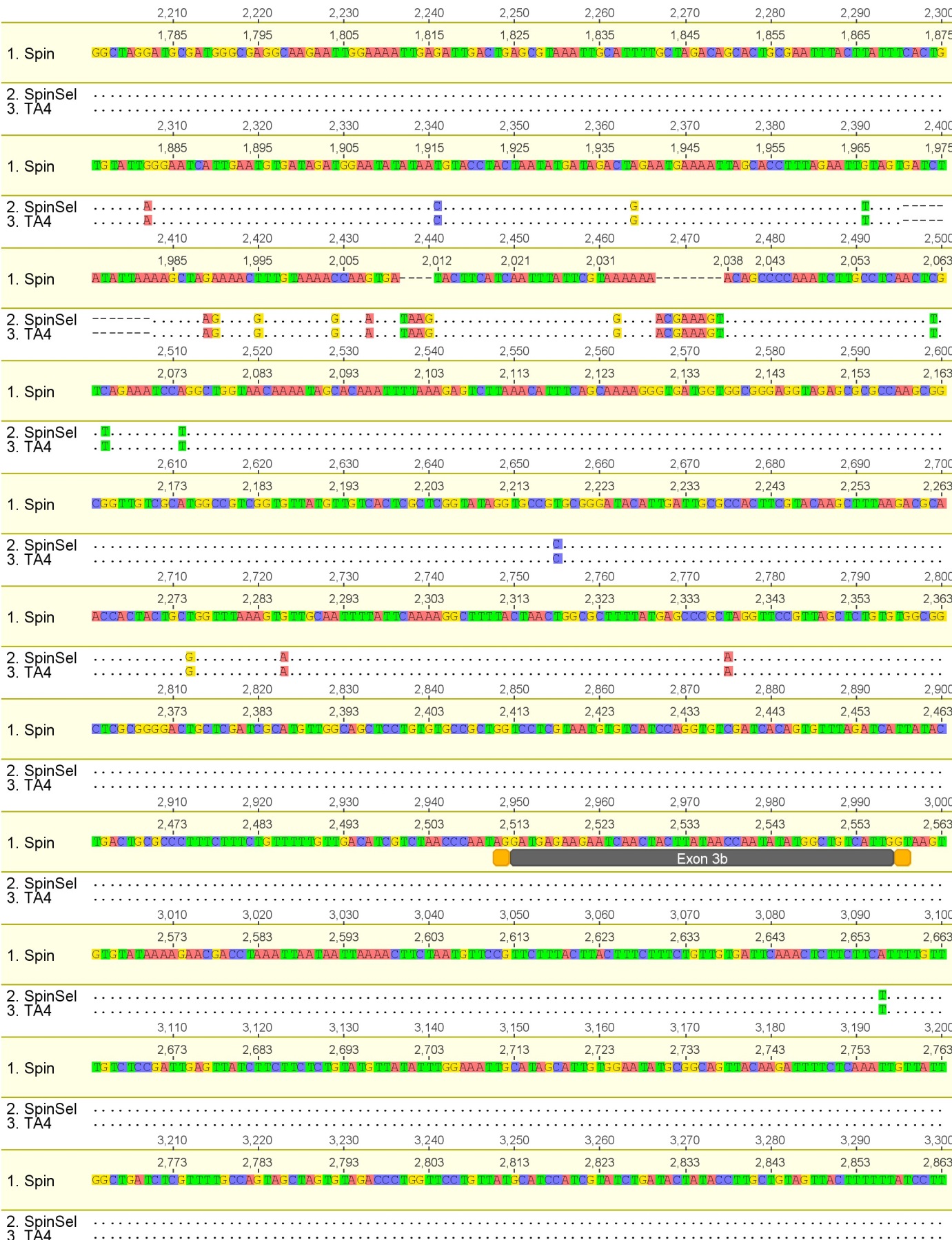


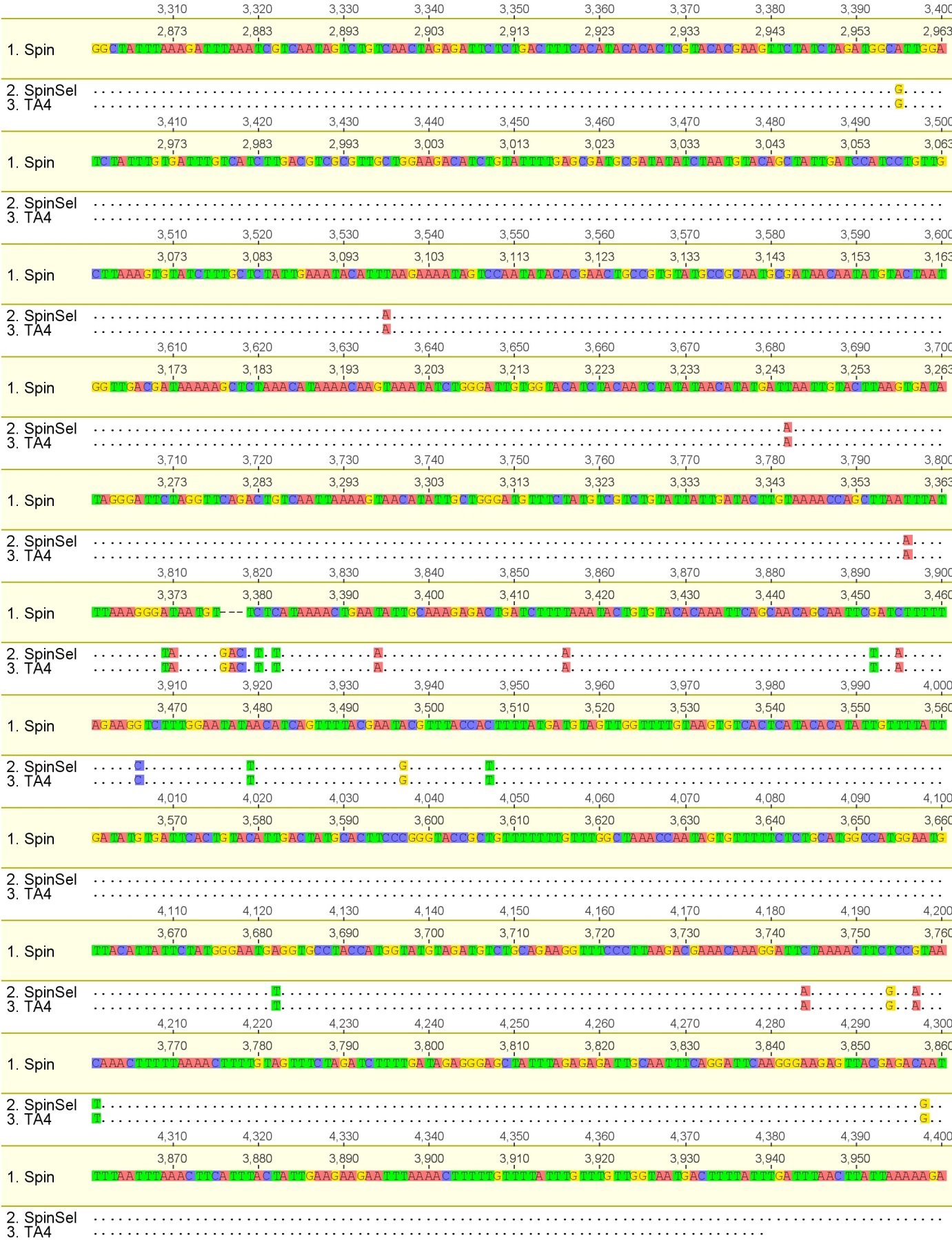

Supplement: Supplementary file 3 — Fig. S3 Alignment of a 4000 bp genomic sequence encompassing the Taα6 exon 3 cluster from the Spin, SpinSel and TA4 strains. [file MEC-25-5692-s003.docx]
