# Supplementary material for: Insecticide resistance mediated by an exon skipping event
Source: Mol Ecol. 2016 Nov 2;25(22):5692–704. doi: 10.1111/mec.13882 (PMC5111602; doi:10.1111/mec.13882)
Supplement: Supplementary file 4 — Fig. S4 Amino acid alignment of the exon 2–4 region of Taα6 from 21 sequenced clones generated from four different Tuta absoluta spinosad susceptible strains (TA1, TA3, TA4, GA). [file MEC-25-5692-s004.docx]

Figure S4. Amino acid alignment of the exon 2-4 region of *Taα6* from 21 sequenced clones generated from four different *T. absoluta* spinosad susceptible strains (TA1, TA3, TA4, GA).
