# Supplementary material for: Insecticide resistance mediated by an exon skipping event
Source: Mol Ecol. 2016 Nov 2;25(22):5692–704. doi: 10.1111/mec.13882 (PMC5111602; doi:10.1111/mec.13882)

Figure S5. Alignment of contigs encoding suppressor of white-apricot from two de novo *T. absoluta* transcriptome assemblies. The top two sequences (comp150190_c0_seq2 and comp72316_c0_seq4) were identified as downregulated in the SpinSel strain and contain a 15bp insertion in the 5'UTR.


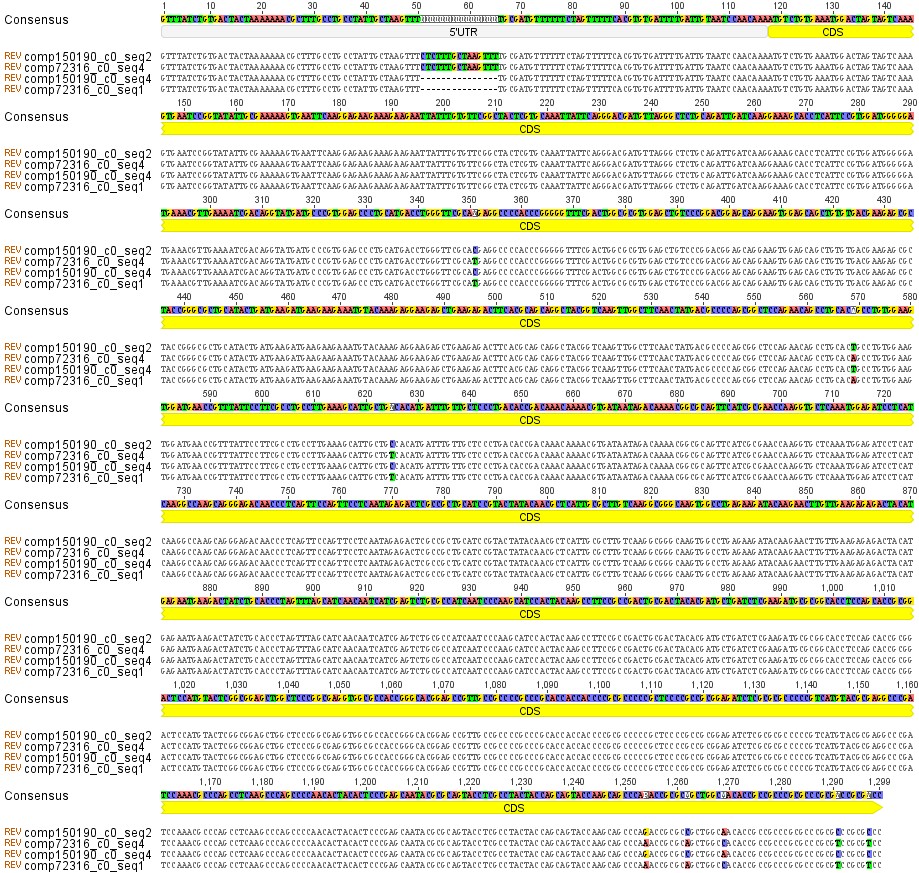

Supplement: Supplementary file 5 — Fig. S5 Alignment of contigs encoding suppressor of white apricot from two de novo Tuta absoluta transcriptome assemblies. [file MEC-25-5692-s005.docx]
