# Supplementary material for: Insecticide resistance mediated by an exon skipping event
Source: Mol Ecol. 2016 Nov 2;25(22):5692–704. doi: 10.1111/mec.13882 (PMC5111602; doi:10.1111/mec.13882)
Supplement: Supplementary file 6 — Table S1 Exon three usage in sequenced clones of Taα6 in the Spin and SpinSel strains. [file MEC-25-5692-s006.docx]

**Supplementary Table 1. Exon three usage in sequenced clones of *Taα6* in the Spin and SpinSel strains.**

| Strain | Exon 3a | Exon 3b | Neither | Total |
| --- | --- | --- | --- | --- |
| Spin | 9 | 6 | 3 | 18 |
| SpinSel | 0 | 0 | 17 | 17 |
