# Supplementary material for: Insecticide resistance mediated by an exon skipping event
Source: Mol Ecol. 2016 Nov 2;25(22):5692–704. doi: 10.1111/mec.13882 (PMC5111602; doi:10.1111/mec.13882)
Supplement: Supplementary file 7 — Table S2 Larval mortality of F2 hybrids of the Spin and SpinSel strains to a discriminating dose of spinosad (120 mg/L). [file MEC-25-5692-s007.docx]

**Supplementary Table 2. Larval mortality of F2 hybrids of the Spin and SpinSel strains to a discriminating dose of spinosad (120 mg L-1).**

| Concentration | Replicate | No. larvae | Alive | Dead | %Mort |
| --- | --- | --- | --- | --- | --- |
| 0 | C1 | 8 | 8 | 0 | 0 |
| 0 | C2 | 7 | 7 | 0 | 0 |
| 0 | C3 | 8 | 8 | 0 | 0 |
| 0 | C4 | 7 | 7 | 0 | 0 |
| 120 | T1 | 8 | 2 | 6 | 75% |
| 120 | T2 | 6 | 1 | 5 | 83% |
| 120 | T3 | 7 | 4 | 3 | 43% |
| 120 | T4 | 6 | 1 | 5 | 83% |
| 120 | T5 | 7 | 3 | 4 | 57% |
| 120 | T6 | 8 | 2 | 6 | 75% |
| 120 | T7 | 8 | 2 | 6 | 75% |
| 120 | T8 | 8 | 2 | 6 | 75% |
| 120 | T9 | 8 | 2 | 6 | 75% |
| 120 | T10 | 8 | 2 | 6 | 75% |
| 120 | T11 | 8 | 4 | 4 | 50% |
| 120 | T12 | 7 | 3 | 4 | 57% |
| 120 | T13 | 8 | 5 | 3 | 38% |
| 120 | T14 | 6 | 3 | 3 | 50% |
| 120 | T15 | 7 | 2 | 5 | 71% |
