# Supplementary material for: Insecticide resistance mediated by an exon skipping event
Source: Mol Ecol. 2016 Nov 2;25(22):5692–704. doi: 10.1111/mec.13882 (PMC5111602; doi:10.1111/mec.13882)
Supplement: Supplementary file 11 — Table S6 Expression of isoforms encoding suppressor of white apricot in the Tuta absoluta transcriptome. [file MEC-25-5692-s011.docx]

**Table S6. Expression of isoforms encoding suppressor of white apricot in the *T. absoluta* transcriptome.** The fragment raw counts of each isoform generated by RSEM for the three biological replicates of the Spin and SpinSel strains sequenced are shown.

| Contig ID | Description | Fragment raw counts | | | | | |
| --- | --- | --- | --- | --- | --- | --- | --- |
|  |  | Spin1 | Spin2 | Spin3 | SpinSel1 | SpinSel2 | SpinSel3 |
| comp150190_c0_seq2 | protein suppressor of white apricot-like | 39.9 | 15.6 | 18.9 | 0 | 0 | 0 |
| comp150190_c0_seq4 | protein suppressor of white apricot-like | 9.9 | 50.1 | 45.1 | 33.2 | 34.9 | 22.3 |
| comp72316_c0_seq1 | protein suppressor of white apricot-like | 38.9 | 155.1 | 199.0 | 122.2 | 138.3 | 94.0 |
| comp72316_c0_seq4 | protein suppressor of white apricot-like | 117.1 | 74.3 | 44.5 | 0 | 0 | 0 |
