# Supplementary material for: Insecticide resistance mediated by an exon skipping event
Source: Mol Ecol. 2016 Nov 2;25(22):5692–704. doi: 10.1111/mec.13882 (PMC5111602; doi:10.1111/mec.13882)
Supplement: Supplementary file 12 — Table S7 Sequences of primers used in this study. [file MEC-25-5692-s012.docx]

**Table S7. Sequences of primers used in this study**.

| Name of Primer | Location | Sequence (5'- 3') | Purpose |
| --- | --- | --- | --- |
| Tuta_nAchR_267R | nAChR α6 exon 4 | ACACCTCCATACTCGCTGTCGTTCC | Reverse primer for RACE to obtain 5' UTR |
| Spoda6F3 | nAChR α6 exon 1 | TGCCCGTRTCGGAGCAAG | Amplification of first half of nAChR |
| Tuta_nAChR_mid_R1 | nAChR α6 exon 7 | GAGTCTGGTGGCAGTGTA | Amplification of first half of nAChR |
| Tuta_nAChR_QR1 | nAChR α6 exon 7 | AACACATGGCACGATCAGGT | Amplification of first half of nAChR (nested PCR) |
| Tuta_nAChR_mid_F1 | nAChR α6 exon 6 | GGAGGCGATTTATCAGACT | Amplification of second half of nAChR |
| Tuta_nAChR_R1 | nAChR α6 exon 12 | AATAGTGTGAACACGAACAGG | Amplification of second half of nAChR |
| Tuta_nAChR_R2 | nAChR α6 exon 11 | ACCTGTCAACAACCATCGC | Amplification of second half of nAChR (nested PCR) |
| Tuta_nAChR_mid_F2 | nAChR α6 exon 6 | TGGCGAATGGTATTTGATAGG | Amplification of second half of nAChR (nested PCR) |
| Tuta_nAChR_ex3a_GSP1 | nAChR α6 exon 3a | CAAATTAAGCCAGACGTTTGTAGTCAGGAT | Genome walking to obtain intron 2 sequence |
| Tuta_nAChR_77F | nAChR α6 exon 2 | GAGCGAGCCWTTAGAGGTCAAGTTCGG | Amplification of intron 2 |
| Tuta_nAChR_In2_377R | nAChR α6 intron 2 | CGTTCTGCGGCTATGAGCTTTCAACCTGA | Amplification of intron 2 |
| Tuta_nAChR_ex3aF | nAChR α6 exon 3a | CAAATCCTGACTACAAACGTCTGG | Amplification of intron 3 |
| Tuta_nAChR_296R | nAChR α6 exon 4 | GCTTGTTTGGTGTGATGCGAACGTCC | Amplification of intron 3 |
| Tuta_nAChR_InR2 | nAChR α6 intron 3b | AGAGTAATGCCTGTAGCTTT | Internal sequencing primer |
| Tuta_nAChR_InSeqF1 | nAChR α6 intron 2 | TGTTTGCCAGATGTGGCGT | Internal sequencing primer |
| Tuta_nAChR_InseqF2 | nAChR α6 intron 2 | CAGACTAGAGATTAAACTTACCT | Internal sequencing primer |
| Tuta_nAChR_InseqR1 | nAChR α6 intron 2 | TACTCCACAAGGGATATATGTAT | Internal sequencing primer |
| Tuta_nAChR_InseqR2 | nAChR α6 intron 2 | AGAGTAATGCCTGTAGCTTT | Internal sequencing primer |
| Tuta_nAChR_InF3 | nAChR α6 intron 3a | CTCGTAATGTGTCATCCAG | Internal sequencing primer |
| Tuta_nAChR_InseqF3 | nAChR α6 intron 3b | AGTCCAATATACACGAACTG | Internal sequencing primer |
| Tuta_nAChR_InseqR3 | nAChR α6 intron 3b | GTGATATAGGGATTCTAGGT | Internal sequencing primer |
| 47101seq2F | integrator complex subunit 12 | GCAGACAGATTGCTGTCCAA | qPCR of potential splice factors |
| 47101seq2R | integrator complex subunit 12 | TGCCCATGTCATTGTCACTT | qPCR of potential splice factors |
| 47101seq2F2 | integrator complex subunit 12 | AAGCGTCGAGCTCTAAAACG | qPCR of potential splice factors |
| 47101seq2R2 | integrator complex subunit 12 | TAGCAAGTCGAAGGGCAACT | qPCR of potential splice factors |
| 42486seq8F | rna-binding protein 1 | GCTACCGTGAATGGGACCTA | qPCR of potential splice factors |
| 42486seq8R | rna-binding protein 1 | ATCTTCAGCGTCGCGTATGT | qPCR of potential splice factors |
| 42486seq8F2 | rna-binding protein 1 | GAATGGGACCTATCCTGCAA | qPCR of potential splice factors |
| 42486seq8R2 | rna-binding protein 1 | CGCGTATGTTTCCGTATTTG | qPCR of potential splice factors |
| 57733seq7F | integrator complex subunit 4 | TGTGAAATTGTTGGGGGATT | qPCR of potential splice factors |
| 57733seq7R | integrator complex subunit 4 | GTCGTAGAGTGTCGCGTTGA | qPCR of potential splice factors |
| 57733seq7F2 | integrator complex subunit 4 | CGCTGGACTTCCTAGTGGAC | qPCR of potential splice factors |
| 57733seq7R2 | integrator complex subunit 4 | CCTCCAAAGCACCCAAGATA | qPCR of potential splice factors |
| 67725seq2F | u11 u12 small nuclear ribonucleoprotein | TCCAGGCAGTACACAACACC | qPCR of potential splice factors |
| 67725seq2R | u11 u12 small nuclear ribonucleoprotein | CGTCAGCTCCAGGTATCTCC | qPCR of potential splice factors |
| 67725seq2F2 | u11 u12 small nuclear ribonucleoprotein | GGGAGGCGTCTGAAACTAGA | qPCR of potential splice factors |
| 67725seq2R2 | u11 u12 small nuclear ribonucleoprotein | ATCCCTGTCGTCTCGATGTC | qPCR of potential splice factors |
| 62457seq2F | gem-associated protein 5 | AAAGGGGAGGAGCACAAACT | qPCR of potential splice factors |
| 62457seq2R | gem-associated protein 5 | GCACGTAGGCCTCCTTGTAG | qPCR of potential splice factors |
| 62457seq2F2 | gem-associated protein 5 | CCCGCAGTCTCCTTCAAATA | qPCR of potential splice factors |
| 62457seq2R2 | gem-associated protein 5 | AGTTTGTGCTCCTCCCCTTT | qPCR of potential splice factors |
| 72316seq4F | protein suppressor of white apricot | CTGCCTTACGAATCCAGCTC | qPCR of potential splice factors |
| 72316seq4R | protein suppressor of white apricot | GGCAGTGTGTTGTTGGTCAC | qPCR of potential splice factors |
| 72316seq4F2 | protein suppressor of white apricot | ATTTGTTGCTCCCTGACACC | qPCR of potential splice factors |
| 72316seq4R2 | protein suppressor of white apricot | TGGAACTGAGGGTTGTCTCC | qPCR of potential splice factors |
| 72316seq4F3 | protein suppressor of white apricot | GATTGCGACGACACATCATC | qPCR of potential splice factors |
| 72316seq4R3 | protein suppressor of white apricot | ACGTAGTCGGCCATTTTGTC | qPCR of potential splice factors |
