# Supplementary material for: Insecticide resistance mediated by an exon skipping event
Source: Mol Ecol. 2016 Nov 2;25(22):5692–704. doi: 10.1111/mec.13882 (PMC5111602; doi:10.1111/mec.13882)
Supplement: Supplementary file 14 — Table S9 Assembly statistics of Tuta absoluta transcriptomes. [file MEC-25-5692-s014.docx]

**Supplementary table x. Assembly statistics of two *de novo* assembled *T. absoluta* transcriptomes.**

| Assembly number | 1 | 2 |
| --- | --- | --- |
| Samples | Spin (3 reps), SpinSel (3 reps) | Spin (3 reps), Spinsel (3 reps), TA1 (1 sample) |
| Type of sequencing | Illumina (1 lane, 6 samples) | Illumina (2 lanes) |
| Digital normalisation? | no | no |
| Assembly | Trinity | Trinity |
| number of raw sequencing reads | 360809452 | 579621326 |
| # of Contigs in assembly | 162474 | 207,300 |
| # of contigs > 200bp | 162474 | 207,300 |
| # of Components/ isotigs | 72934 | 87062 |
| # of trinity 'genes' | 80714 | 103980 |
| % GC | 40% | 39% |
| Min contig length | 201 | 201 |
| Max contig length | 29,110 | 30845 |
| Mean contig length (bp) | 1059 | 1011 |
| Median contig length (bp) | 537 | 478 |
| N50 | 1967 | 1,979 |
| Blast2Go hits | 57160 | 66,755 |
| Blast2Go hits % | 35% | 32% |
